# The Segmentation Method of Target Point Cloud for Polarization-Modulated 3D Imaging

**DOI:** 10.3390/s20010179

**Published:** 2019-12-28

**Authors:** Shengjie Wang, Bo Liu, Zhen Chen, Heping Li, Shuo Jiang

**Affiliations:** 1 Key Laboratory of Space Optoelectronic Precision Measurement Technology, CAS, Chengdu 610209, China; wang.shengjie@foxmail.com (S.W.); beyondcz@foxmail.com (Z.C.); jingjingyua@163.com (S.J.); 2 University of Chinese Academy of Sciences, Beijing 100049, China; 3 Institute of Optics and Electronics, Chinese Academy of Sciences, Chengdu 610209, China; 4 University of Electronic Science and Technology of China, Chengdu 611731, China; oehpli@uestc.edu.cn

**Keywords:** polarization-modulated, LiDAR, data fusion, target segmentation

## Abstract

To implement target point cloud segmentation for a polarization-modulated 3D imaging system in practical projects, an efficient segmentation concept of multi-dimensional information fusion is designed. As the electron multiplier charge coupled device (EMCCD) camera can only acquire the gray image, but has no ability for time resolution owing to the time integration mechanism, large diameter electro-optic modulators (EOM) are used to provide time resolution for the EMCCD cameras to obtain the polarization-modulated images, from which a 3D image can also be simultaneously reconstructed. According to the characteristics of the EMCCD camera’s plane array imaging, the point-to-point mapping relationship between the gray image pixels and point cloud data coordinates is established. The target’s pixel coordinate position obtained by image segmentation is mapped to 3D point cloud data to get the segmented target point cloud data. On the basis of the specific environment characteristics of the experiment, the maximum entropy test method is applied to the target segmentation of the gray image, and the image morphological erosion algorithm is used to improve the segmentation accuracy. This method solves the problem that conventional point clouds’ segmentation methods cannot effectively segment irregular objects or closely bound objects. Meanwhile, it has strong robustness and stability in the presence of noise, and reduces the computational complexity and data volume. The experimental results show that this method is better for the segmentation of the target in the actual environment, and can avoid the over-segmentation and under-segmentation to some extent. This paper illustrates the potential and feasibility of the segmentation method based on this system in real-time data processing.

## 1. Introduction

The light detection and ranging (Lidar) system can produce a three-dimensional (3D) representation of the scene’s 3D structure, and the array laser radar is considered as a leading technology to obtain high-density 3D spatial information. The current challenge is to have the ability of real-time point cloud data processing, while maintaining high speed and high-resolution 3D imaging [1,2].

Polarization-modulated 3D imaging Lidar utilizes dual electron multiplier charge coupled device (EMCCD) cameras to accumulate the returned-light, whose polarization state is modulated by an electro-optic modulator (EOM) [2]. The depth image is reconstructed using a polarization-modulated image (including cos^2^ modulated image and sin^2^ modulated image) [3]. Its advantage is that it simplifies the data acquisition of remote imaging, so that the polarization-modulated 3D imaging system, known as a flash 3D Lidar, can not only improve the performance of dynamic 3D imaging, but also provide a high-resolution imaging effect (1024 × 1024 pixels or higher). This provides a hardware basis for the point cloud data of the high-resolution field of view in later activities and the processing of point cloud data (such as target point cloud segmentation and target attitude measurement).

However, in practical applications, it is very difficult and time-consuming to extract valuable spatial information from 3D laser imaging data owing to the complex imaging background, the diversity of 3D objects, and the large number of point clouds. The point cloud segmentation refers to the classification of points with the same or similar attributes and adjacent spatial positions in point cloud data into a class through certain methods. It can segment a single frame point cloud or point cloud model. In the last ten years, the commonly used segmentation methods of 3D point cloud laser data mainly include the edge-based segmentation algorithm, region-based segmentation algorithm, and clustering-based segmentation algorithm.

The edge-based segmentation algorithm considers that the mutation of the normal vector or curvature is the boundary of a region, and the region surrounded by the closed boundary is the final segmentation result. The most important thing with the edge-based segmentation algorithm is to find the boundary of the region. The work of [4] builds a symbolic distance function to estimate the average curvature of the point cloud, and uses the 3D active contour model to realize point cloud model segmentation. The shortfall of this method is that, owing to the influence of noise, the edge positioning accuracy of the point cloud model is poor, which makes the segmentation algorithm based on edge inadequate.

The segmentation algorithm based on region contains two ideas. One is the region growth algorithm [5,6]. Select the seed point, and spread it to the neighborhood according to the growth strategy until there is no continuous point set. The other is hierarchical decomposition, assuming that all point sets belong to the same target, octree [7] and KD (k-dimensional) tree [8] are adopted for hierarchical decomposition to obtain the segmentation results of different details. Although this method is widely used in 3D point cloud segmentation and is simple to implement, its robustness is not good. Because of the influence of multiple evaluation criteria of segmentation, the calculation is time-consuming, and thus it is not suitable for real-time imaging data processing.

In the clustering-based segmentation method, the point cloud segmentation is regarded as the classification process of data points with certain characteristic parameters. In the literature [9,10,11], mean-shift clustering, spectral clustering, and fuzzy clustering, respectively, are adopted to realize the segmentation of point cloud models. As well as the depth data segmentation method based on the Gaussian mixture model clustering, the normal vector of 3D point cloud is clustered using the Gaussian mixture model. K-means is also a common processing method for the registration algorithm [12]. Then, the random sampling consistency algorithm is used to perform the plane fitting to each cluster to achieve the plane extraction of scene data. On the basis of the segmentation method of clustering, different clustering leads to different results. The advantage of this method is that it has good robustness and does not need to search points or search areas. However, when the volume of point could data is very large, there will be a large amount of segmentation computation, and continuous boundary points cannot be detected. Therefore, refinement processing is needed after segmentation, which is the reason that this method is not applicable to our 3D imaging system.

In this system, polarization-modulated 3D imaging Lidar is used as array receiver by EMCCD (electron multiplying charge coupled device) [13]. EMCCD is a new type of image sensor with high sensitivity, which can provide high lateral resolution [14]. However, owing to the time integration mechanism, EMCCD can only obtain the intensity diagram, but not the depth diagram of the scene. Fortunately, time-resolved imaging can be achieved by means of polarization modulation, in order to obtain the corresponding distance information of each pixel and to obtain 3D point cloud data in the imaging field. We use the characteristics of the system, based on grayscale map, using the maximum entropy adaptive segmentation method, to complete the initial segmentation of the target, combined with the corrosion treatment of images, binary graphs containing only target information can be obtained accurately. Next, the accurate target coordinate position is obtained in the image, and the mapping relationship between target gray coordinate and depth coordinate is established to obtain the target point cloud after segmentation.

Compared with other segmentation methods, the method in our paper combines the characteristics of the polarization-modulated 3D imaging system. Using EMCCD camera to collect the grayscale image and depth image simultaneously, the processing dimension of point cloud data is reduced to the processing of the gray image, which simplifies the data segmentation algorithm of the array point cloud, and greatly reduces the amount of data processed. This solves the problem of target point cloud segmentation in common specific scenarios. At the same time, the idea of point cloud segmentation can also be adjusted according to the state of experimental targets in different experimental background environments, and it can be applicable to more diverse actual experimental environments. Thus, the problem of target point cloud segmentation can be completed efficiently and accurately.

## 2. Principal of Operation

### 2.1. Polarization-Modulated 3D Imaging System

**Figure 1:**
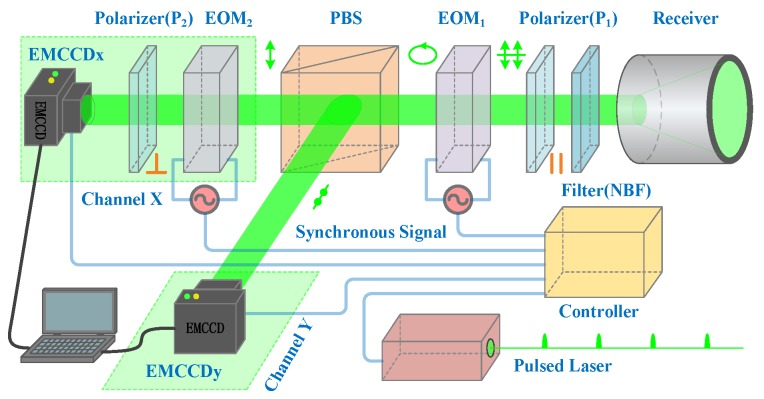
Polarization-modulated imaging framework. Here, the first electro-optic modulator (EOM1) manipulates the polarization state of the returned-light to perform time-resolved imaging, while the second modulator (EOM2) acts as a fast shutter for range-gated imaging. Dual electron multiplier charge coupled device (EMCCD) cameras acquire the polarization-modulated images for 3D reconstruction in channel X and Y, respectively. NBF, narrowband filter; PBS, polarization beam splitting.

The 3D imaging framework for our experiment is illustrated in Figure 1. The pulsed laser works as an illuminator at a low repetition rate (10 Hz), high pulse energy (200 mJ), and short pulse duration (8 ns). A linear-polarized light is emitted towards the scene and then reflected/scattered by the objects. When the returned light enters the receiver, background radiation from sunlight and other sources is removed by a narrowband filter (NBF). Afterwards, a linear polarizer (P1, parallel to the emitted linear-polarized light) is required to isolate other polarized light, leaving only the linear-polarized light parallel to the emitted linear-polarized light. Applying a ramp voltage to the electro-optic modulator (EOM1), phase retardation proportional to the voltage will take place. Consequently, the linear-polarized light is converted into an elliptical-polarized light [15]. With polarization beam splitting (PBS), the elliptical-polarized light is separated into two orthogonal polarized components; that is, p-polarized and s-polarized light. Alternately, we could use twice as many EMCCD cameras to detect both the p-polarized and the s-polarized light. The p-polarized light passes through channel X and arrives at EMCCDx, while the s-polarized light is reflected to channel Y and arrives at EMCCDy.

In the dual cameras structure, the ratio of intensities in one camera versus the other camera provides range information, while the summation of intensities in both cameras provides transverse information. Ultimately, a 3D image of the scene can be produced after 3D reconstruction. The biggest advantage of this technique is that we can use a pair of EMCCD cameras for super-resolution 3D imaging, with which a frame of the 3D image can be reconstructed from a frame of the polarization-modulated images. This advantage causes the 3D imaging framework to act as a flash Lidar system, especially suitable for dynamic 3D imaging, regardless of objects motion or platform motion.

As shown in Figure 1, in order to ensure that the returned-light is modulated and accumulated accurately, synchronous signals are required to manipulate the pulsed laser, electro-optic modulators, and EMCCD cameras properly. Dual EMCCD cameras are connected directly to a computer and then a frame of raw data is acquired for 3D reconstruction during a 0.1 s period, which corresponds to one pulsed-light propagating from the Lidar system to the scene and back. In this case, reconstructing a frame of the 3D image requires emitting only a pulsed-light. Although the frame rate of cameras is 10 Hz, the exposure time is merely 0.32 microseconds (us). Consequently, when an object flies across the scene at a speed of 100 m/s, its transverse displacement is merely 30 um. Such a moving object can be undoubtedly treated as a motionless object.

In the 3D imaging framework, the electro-optic modulator based on the electro-optic effect of crystals is applied to realize time-resolved imaging. When a ramp voltage is applied to the electro-optic modulator in the direction of propagation, phase retardation between the ordinary wave and the extraordinary wave will take place, which is proportional to the applied voltage. Because of the fact that objects located at a different range will result in different roundtrip times, the phase retardation θ can be rewritten as a function related to range.

(1) $$\theta =\pi \cdot \frac{D}{L},\left(0\leq D\leq L\right),$$

where *L* is the gate opening range determined by the duration time of ranged-gate opening, and *D* is the range between the object and the gate beginning range $R_{Base}$. Such phase retardation will lead to different intensity distributions in channel X and Y, respectively [1,16].

(2) $$\left\{ \begin{array}{l} I_{X}=I_{REC}\cos^{2}\left(\frac{\pi }{2}\cdot \frac{D}{L}\right)\\ I_{Y}=I_{REC}\sin^{2}\left(\frac{\pi }{2}\cdot \frac{D}{L}\right) \end{array}\right.,\left(0\leq D\leq L\right),$$

where $I_{REC}$ is the intensity of returned-light and $I_{X}$ and $I_{Y}$ are the intensities of the p-polarized and s-polarized components, respectively. Obviously, the polarization-modulated images derived from Equation (2) contain not only the intensity image, but also range information. Therefore, 3D reconstruction can be implemented according to the above equation. Finally, the range R between the Lidar system and the object can be given by

(3) $$R=R_{Base}+\frac{2L}{\pi }\arctan\left(\sqrt{\frac{I_{Y}}{I_{X}}}\right).$$

Besides, it should be noticed from Equation (4) that the summation of $I_{X}$ and $I_{Y}$ will produce a polarization-demodulated image (conventional gray image) as follows:

(4) $$I_{X}+I_{Y}=I_{REC}.$$

The range resolution, which is directly proportional to the phase resolution, can be obtained from the following expression:

(5) $$\Delta R=\frac{2L}{\pi }\Delta \theta =\frac{2L}{\pi }\arctan\left(\sqrt{\frac{1}{2^{n}}}\right),$$

where Δ*θ* is the phase resolution and *n* is the resolution of the analog-to-digital converter (ADC) in bits. With regard to a 16-bit ADC (*n* = 16), the number of discrete gray-values available in the EMCCD camera is 2^16^ (65,536). In addition, we know that a gate opening range (*L*) of 48 m is designated given a gate opening time of 0.32 μs. Ultimately, the range resolution is approximately 10 cm for our 3D imaging framework.

### 2.2. Image Segmentation Based on Intensity Image

The advantage of this system is that it can obtain the point cloud data and grayscale map of the distant target through a single frame of data. Therefore, the system has a good application prospect in the field of three-dimensional data acquisition and real-time processing of high-speed moving targets such as spacecraft in-orbit service and projectile attitude estimation. The characteristic of this kind of scene is that the target has the global maximum entropy in the image and the background is relatively simple.

**Figure 2:**
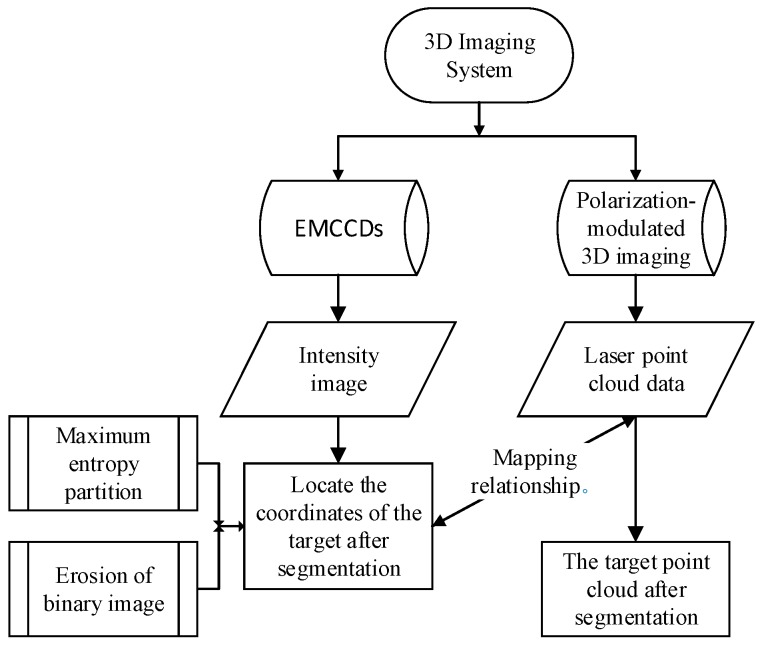
Proposed of segmentation methodology.

In order to achieve real-time target’s point cloud data processing, how to segment the target from the complex background quickly and accurately is a very critical step. An efficient segmentation method of 3D point cloud based on sensor information fusion for polarization-modulated 3D imaging LiDAR is proposed in this paper. Compared with the traditional clustering segmentation method, this method has the advantages of fast processing speed and high segmentation accuracy. Firstly, target segmentation is completed based on gray image processing. Then, the mapping relationship between gray image and point cloud data is established. Finally, the target pixel coordinates are obtained after segmentation in the grayscale image, finding the corresponding position coordinates of the point cloud, and thus completing the 3D point cloud segmentation of the target. As a result, computational complexity is reduced and memory size is saved simultaneously, especially when the point cloud data volume is large. The specific process is shown in Figure 2.

In this system, polarized 3D imaging Lidar is used as an array receiver by two EMCCDs. EMCCD is a new type of image sensor with high sensitivity, which can provide a high horizontal resolution and obtain a 1024 × 1024 pixel intensity image. On the basis of this advantage, we adopted the maximum entropy segmentation method to process the captured high-resolution light intensity map, which can complete the initial segmentation of the target in the complex background quickly and effectively.

Entropy is used to measure the uniformity of a distribution, and higher entropy means more uniform distribution [17]. An image with 1024 × 1024 pixels can be interpreted as information containing 1024 × 1024 symbols; the values of each symbol are independently obtained from different gray-values in a finite range of K (0–255). The digital image is modeled as a random signal processing, which means that the probability of occurrence of each grayscale in the image grayscale must be known, namely *p*(*g*), then the solution formula of information entropy in digital images is as follows:

(6) $$H=-\int_{-\infty }^{+\infty }p(g)\log[p(g)]dx.$$

In the grayscale image, set the probability that the grayscale value is *i* as *p_i_*, and the threshold value as *T* (0 ≤ *T* ≤ *K* − 1), so the grayscale value of background is *B*, and the grayscale value of foreground is *F*.

Therefore, the probability of each gray level in the foreground and background can be calculated as follows:

background *B*:

(7) $$P_{i}=\frac{p_{i}}{p_{T}},i=0,1,2,\ldots ,T,$$

in which $p_{T}=\sum_{i=0}^{T}p_{i}$

foreground *F*:

(8) $$P_{i}=\frac{p_{i}}{1-p_{T}},i=T+1,T+2,\ldots ,K-1.$$

The information entropy of background *H_B_* and foreground *H_F_* is, respectively,

(9) $$\begin{array}{l} H_{B}=-\sum_{i=0}^{T}P_{i}\log(P_{i})\\ H_{F}=-\sum_{i=T+1}^{K-1}P_{i}\log(P_{i}) \end{array}$$

Calculate the total entropy of images under all segmentation thresholds, find the maximum entropy, and take the segmentation threshold corresponding to the maximum entropy as the final threshold; pixels in the image whose grayscale is greater than this threshold are used as foreground, and otherwise as background. Traverse the exhaustion threshold *T* from 0 to 255, get the threshold *T* that maximizes *H_B_*+ *H_F_*, and the threshold *T* is the gray image segmentation point obtained by the maximum entropy algorithm [18]:

(10) $$T=\arg\max(H_{B}+H_{F}).$$

After maximum entropy segmentation, binary image $I_{binary}$ is generated. However, although the segmentation of the target can be completed efficiently only through maximum entropy, it is inevitable that other background objects will affect the segmentation effect in practical application, and the binary graph of the target cannot be retained purely. This will affect the subsequent extraction of target coordinates through the segmented binary graph. To this end, we adopted the image morphological erosion algorithm on binary images, and eliminated the noise interference to the maximum extent under the loss of a small number of target edges, so as to obtain only the binary-graph coordinates of the target [19].

The set of 1024 × 1024 pixels in binary image $I_{binary}$ is divided into the binary set $I_{Tar}$ and structural element (SE) after maximum entropy segmentation, using SE to erode $I_{Tar}$:

(11) $$I_{Tar}\Theta SE=\{I_{binary}|(SE)I_{binary}\subseteq I_{Tar}\}.$$

Let the structural element *SE*, which is originally located at the origin of the image, move across the entire binary graph plane. If *SE* can be completely contained in $I_{Tar}$ when the origin of *SE* is translated to the point of $I_{binary}$, then the set composed of all such points in the binary image $I_{binary}$ is the image eroded by *SE* on $I_{Tar}$. In this paper, 3 × 3 disc-shaped structural elements whose origins are located in the center are used for corrosion operation. Through the erosion of the binary image and filtering of the influence of noise outside the target, the binary image containing only the target can be obtained in the binary graph $I_{binary}$, and then the accurate target coordinate can be obtained.

In this paper, the polarization-modulated 3D imaging system realizes time resolution imaging by means of polarization modulation, to obtain the corresponding distance information of each pixel and the depth map of the imaging field. In this way, two-channel EMCCDs not only serve as a receiver of light intensity in this system, but also can realize the time resolution ability to complete ranging, and obtain the point cloud information of a high-resolution plane array field of view. More importantly, the intensity maps correspond to the pixels in the depth maps. By establishing the coordinate mapping relationship between the two, the coordinate information of the target can be located directly through the binary graph containing only the target, the coordinate position can be mapped to the three-dimensional point cloud data, and the target point cloud data in the three-dimensional information can be obtained directly.

## 3. Experimental Results

Owing to the complex composition of the experimental system and the large volume of the laser device, the 3D imaging system can only be fixed on the optical platform of the laboratory at present. Limited by the experimental environment, the experimental target is placed on the roof of the residential building about 1 km away from the imaging system.

**Figure 3:**
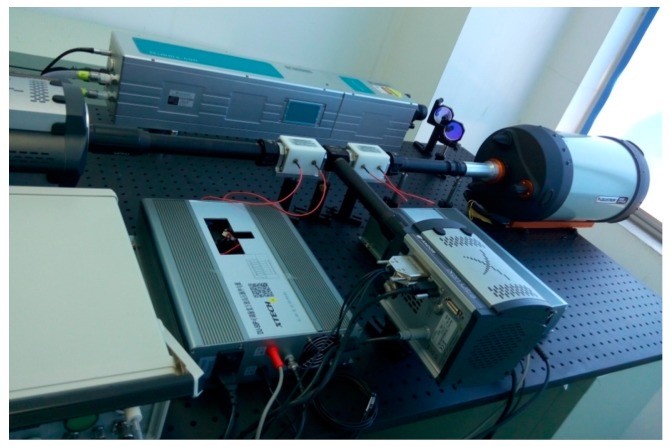
Experimental setup of super-resolution 3D imaging.

The experimental setup for 3D imaging is shown in Figure 3, and the intensity image and full depth image will be reconstructed in a single pulsed cycle. During the course of work, dual EMCCDs with a resolution of 1024 × 1024 pixels are applied to accumulate the returned-lights in channel X and Y, respectively. And the parameters for 3D imaging system are shown in Table 1.

The proposed segmentation approach can be applied for the laser data obtained by polarization-modulated 3D imaging Lidar. However, the threshold defined in this algorithm should be adjusted according to the laser data sets of segmentation targets in different environments’ backgrounds.

**Figure 4:**
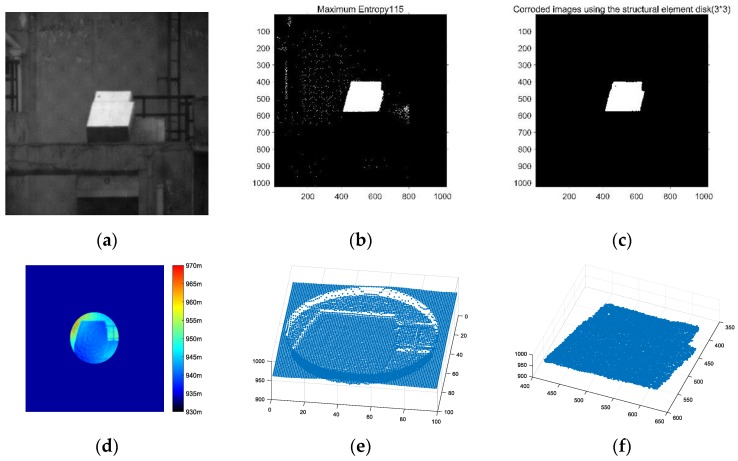
Polarization-modulated 3D imaging Lidar data: (**a**) intensity image; (**b**) maximum entropy segmentation effect; (**c**) binary diagram effect after erosion; (**d**) depth image; (**e**) point clouds in the field of view before segmentation; (**f**) segmentation of target point cloud.

The Lidar system uses an EMCCD camera as a detector so that we can capture 3D features and grayscale images of objects in the field of view simultaneously, as shown in Figure 4a,d. The feature of the system makes it possible for us to complete point cloud segmentation through image processing. The reliability and robustness of the newly developed technique will be examined through the following experiments.

Figure 4 shows the processing effect images of each stage in the segmentation method flow chart in Figure 2. Figure 4a is the intensity image of polarization-modulated 3D imaging of a carton, which acts as our experiment target. 

On the basis of the characteristics of the high discrete gray value of EMCCD, an adaptive maximum entropy segmentation method is adopted to significantly separate the target from the complex background. According to the binary graph effect after maximum entropy segmentation, as shown in Figure 4b, although the target can be segmented, there is still interference caused by the grayscale of other objects, and the pixel coordinates of the target cannot be accurately located. 

Then, the morphological erosion algorithm is used on the binary image after maximum entropy segmentation. The processing result is shown in Figure 4c, which can perfectly retain the binary image of the target, eliminate the interference of pixels caused by other objects’ grayscale, accurately locate the coordinates of the target, and segment the target from the grayscale image. 

The depth map obtained by Lidar is shown in Figure 4d, in which the color represents the distance of each pixel. It can be known that the distance between the target and the 3D imaging system is about 940–945 m. After converting the depth map into a point cloud map, it can be seen from Figure 4e that, before we separate the target from the background, the point cloud in the field of view includes not only our target, but also the point cloud data of other objects such as railings and walls.

By mapping the target coordinate value after image segmentation to the corresponding depth graph, the target point cloud data information can be obtained, and the target point cloud can be accurately and effectively segmented from the complex background. The segmentation effect of the target point cloud is shown in Figure 4f, where the *X*-axis (350–600 pixels) and *Y*-axis (400–650 pixels) coordinates represent the pixel coordinate position in the target’s field of view, respectively, and the *Z*-axis coordinate (900–1000 m) represents the distance information of each pixel of the target from the 3D imaging system.

The comparison of derived segmentation results with segmentation results (Figure 4f) and image data (Figure 4a) proves the feasibility and robustness of this method in a complex background by establishing the mapping relationship between the grayscale image and point cloud data through the target segmentation results of the grayscale image.

In order to demonstrate the advantages of accuracy and efficiency of remote target cloud segmentation of this method, based on the same experimental data, we compare this method with the traditional point cloud segmentation methods mentioned in the introduction.

In the process of point cloud segmentation, the number of point clouds occupied by the target will affect the accuracy and time of segmentation. Generally speaking, the more points used to represent the target, the higher the segmentation accuracy of the algorithm, but at the same time, the larger the computation, the longer the algorithm takes. Therefore, in the evaluation of the segmentation effect, the number of point cloud, segmentation time, and accuracy of segmentation are the standard to judge the quality of point cloud segmentation.

**Figure 5:**
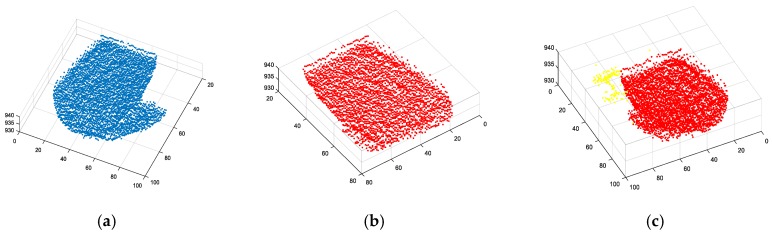
The segmentation effect of the traditional segmentation method on experimental data. (**a**) Segmentation algorithm based on edge detection; (**b**) segmentation algorithm based on region growth; (**c**) segmentation algorithm based on Euclidean clustering.

The segmentation method of edge detection can quickly calculate the edge information of the target and complete the target segmentation. However, this method is not able to separate the front and rear boxes of the stacked ones (as shown in Figure 5a), and is not sensitive to the curved surface with a slow shape change or large rounded corner radius. In the presence of noise, excessive segmentation is inevitable.

When we use the segmentation algorithm based on region growth, the result is shown in Figure 5b. We can see that the main part of the target is segmented, but because of the influence of noise, excessive segmentation occurs. In terms of time consumption, with the increase of the number of point clouds, this algorithm will take longer. When the number of point clouds of the target exceeds 70% of the total point clouds of the detector, it will take more than 20 s, which obviously cannot be used for real-time data processing.

The segmentation method based on Euclidean clustering is ideal for separating objects and railings, as shown in Figure 5c. However, because of the influence of noise in the actual experiment, the phenomenon of excessive segmentation still appears. In the segmentation of the target bin, the point cloud of the rear bin is also segmented. However, the objects with few point clouds such as railings are not segmented enough. Nevertheless, although the effect of the clustering algorithm is relatively ideal in point cloud segmentation, this algorithm needs to first accurately calculate the attributes of point cloud data, so as to classify the best effect according to the attributes, which leads to the exponential increase of algorithm running time as the number of point clouds increases. When the number of points involved in the calculation is too high, the algorithm can even take more than 100 s.

**Figure 6:**
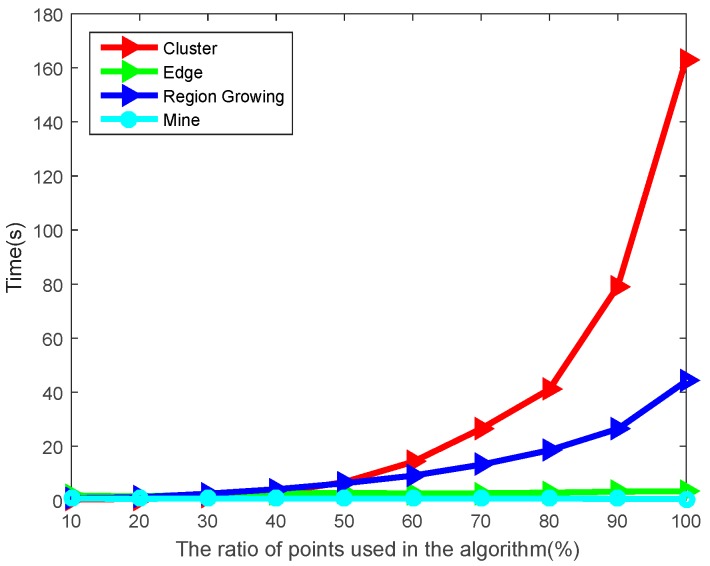
The time of different segmentation algorithms under different cloud numbers.

In the time-consuming comparison of the four algorithms, we used the ‘downsample’ function in MATLAB (MathWorks company, Natick, MA, USA) to sample the point cloud and image data by 10–100%, and compared the time consumption of the data on the target in different states. The computer configuration used in the calculation is Intel(R) (Intel Corporation, Santa Clara, CA, USA), Core(TM), i5-4200CPU@2.80GHz, 4GB RAM, and the programming environment is Matlab R2015b. We can intuitively see the time difference between different methods for the same data in Figure 6.

As can be seen from the green curve in Figure 6, the time consumption of the edge segmentation algorithm is relatively stable. With the increase of the number of point clouds, the algorithm is stable around 3 to 5 s, which not only has low segmentation accuracy, but also fails to meet the real-time demand. The region growth segmentation algorithm is represented by the blue curve in Figure 6; when the number of point clouds is small, the algorithm is more efficient, but as the number of point clouds increases, it takes longer and is not suitable for real-time data processing. The red curve represents the time of the clustering segmentation algorithm; although the segmentation effect is acceptable, it takes too long and can only be used for post-point-cloud processing. The image-based segmentation method proposed in this paper is not affected by 3D data, and only needs to process 2D data, so the time complexity of the algorithm is lower. The cyan curve in Figure 4 shows that the time of our algorithm is stable, and the average time is only 0.72 s; the application of this algorithm in the subsequent point cloud real-time data processing can be guaranteed.

## 4. Discussion

Benefitted by the neoteric polarization-modulated 3D imaging method, we took advantage of the EMCCD camera in the system as a planar array detector, and simultaneously obtained the grayscale image of the field of view and the 3D point cloud data in the process of one frame shooting. On the basis of this feature, the correlation model between the laser ranging value and image pixel is established. The idea of point cloud segmentation is proposed to reduce the dimension of 3D data processing to 2D data processing. By this method, the algorithm complexity can be reduced by one order of magnitude, and the efficiency of point cloud segmentation can be greatly improved. Moreover, with the high horizontal resolution of the EMCCD camera, the segmentation effect is also very impressive.

The system is intended to be used in the field of in-orbit spacecraft recognition, inertial navigation, and robot vision, among others. When the information entropy of the target is the maximum in the application scenario, the image processing method mentioned in this paper is very effective and applicable. When applied to the recognition and segmentation of rigid bodies like vehicles, Hough transform and other image processing methods may be a better choice.

The main innovation of this paper is the fusion of multidimensional data relationships of the detector. When we establish the coordinate relation between the point cloud and the image, we can reduce the dimension of 3D data processing to image processing, so as to be applicable to more scenes and application requirements.

## 5. Conclusions

Extraction of spatial target information from laser point cloud with a complex background requires a reliable segmentation approach. Although many technologies have been developed for this kind of data segmentation, there are a few methods to deal with the laser point cloud data set collected by the polarization-modulated 3D imaging system with high accuracy and in real time. In addition, when the polarization imaging system is applied in practice, there will be some practical problems such as noise interference and systematic errors, which will reduce the segmentation accuracy of traditional clustering segmentation methods, which are time-consuming and not suitable for real-time point cloud data processing.

This paper introduces a new segmentation technique for multi-sensor data fusion. The technique can be applied to polarization-modulated 3D imaging laser data sets with different target density and noise levels when the target has global maximum entropy in the intensity image. Firstly, the parameters of the probability density function are estimated in the grayscale image, and then the maximum likelihood ratio test is used for segmentation. The iterative algorithm is used to improve the accuracy of segmentation, which can be applied to target segmentation of the gray image with an arbitrary probability density function. Finally, the mapping relationship between the gray information and depth information collected by EMCCDs in polarization-modulated 3D imaging system is established. In this way, the target segmentation coordinates completed in the grayscale map can be mapped to the 3D target point cloud, in order to obtain the 3D target point cloud data after segmentation.

The main advantage of this segmentation method is that it is suitable for the polarization-modulated 3D imaging system, which can greatly reduce the data volume and significantly improve the robustness. Meanwhile, this method avoids the problems of poor segmentation accuracy and large segmentation error caused by the error of point cloud data. This method has great potential and can be applied to different environments with a little modification, providing a possibility for real-time processing of point cloud data.

## Figures and Tables

**Table 1:** Parameters for 3D Imaging System.

System Parameters	Value
Wavelength	532 nm
Pulse Energy	200 mJ
Pulse Duration	8 ns
Frame Rate	10 Hz
Resolution	1024 × 1024 pixels
Aperture	200 mm
